# Green Coffee Bean Extract Assisted Facile Synthesis of Reduced Graphene Oxide and Its Dye Removal Activity

**DOI:** 10.1002/gch2.202300247

**Published:** 2023-12-21

**Authors:** A.B.M. Nazmul Islam, Prianka Saha, Md. Emran Hossain, Md. Ahsan Habib, Kaykobad Md. Rezaul Karim, Md. Mahiuddin

**Affiliations:** ^1^ Chemistry Discipline Khulna University Khulna 9208 Bangladesh; ^2^ Physics Discipline Khulna University Khulna 9208 Bangladesh

**Keywords:** adsorption, cationic dye, chemisorption, exfoliation, green coffee bean, green synthesis, reduced graphene oxide

## Abstract

To discharge the colored effluents from industries there needs to be effective and affordable treatment options. Adsorption using reduced graphene oxide (rGO) as an adsorbent is a prominent one. In this study, green coffee bean extract (GCBE) is utilized as a safe reducing agent for the reduction of graphene oxide (GO) to synthesize rGO. The formation of rGO is confirmed by a new peak in the UV–vis spectra at 275 nm and a diffraction peak in the XRD patterns at 22°. The effective formation of rGO is further substantiated by a change in the GO peak's properties in the FTIR, EDX, and Raman spectra and a weight loss change in TGA. The SEM and TEM analyses demonstrate the effective production of the nano‐sheets of rGO having exfoliated and segregated in a few layers. Furthermore, the obtained rGO exhibited outstanding efficacy in wastewater cleanup, effectively adsorbing MB as a prototype organic dye. The kinetics and isotherm study suggested that the adsorption leads by the chemisorption and monolayer formation on the homogeneous surface of rGO. The maximum adsorption capacity is found to be 89.3 mg g^−1^. This process offers a fresh opportunity for the economical and safe production of rGO for wastewater treatment.

## Graphical abstract

As a safe reducing agent, green coffee bean extract converts GO to rGO and then uses the obtained rGO to remove methylene blue through adsorption.

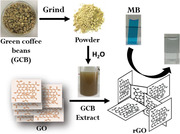

## 1 Introduction

In the post‐graphene discovery era, reduced graphene oxide (rGO) has gained the greatest research focus due to its distinctive physicochemical properties including optical transparency, amazing carrier mobility, and large surface area. The effective application of rGO both alone and in composite form in a variety of industries, such as battery manufacturing and saltwater desalination, further sparked interest in the sector.^[^ ^1^, ^2^, ^3^, ^4^, ^5^, ^6^ ^]^ Most often, the aqueous phase chemical reduction has been used as one of the cheaper and simpler methods to produce more stable rGO.^[^ ^2^, ^4^, ^5^, ^7^, ^8^, ^9^, ^10^, ^11^ ^]^ Feng et al. used a sodium‐ammonia as the reducing agent for the reduction of GO and got high hole mobility of 123 cm^2^ Vs^−1^ with a combined low sheet resistance (≈350 Ω per square with ≈80% transmittance).^[^ ^4^ ^]^ After Stankovic et al., Chua et al.^[^ ^7^, ^8^ ^]^ also used hydrazine‐hydrate as the reducing agent to reduce GO and get exfoliated sheets of rGO. Shin et al. used both sodium borohydride and hydrazine–hydride for the reduction of GO and prepared the transparent conducting film of rGO. They also found that sodium borohydride‐mediated rGO film has much lower sheet resistance than hydrazine‐mediated rGO film.^[^ ^2^ ^]^ Zhu et al. used glucose for the reduction of GO and the obtained rGO showed good electrocatalytic activity toward catecholamines.^[^ ^3^ ^]^ Zhang et al., De Silva et al., and Gao et al.^[^ ^12^, ^13^, ^14^ ^]^ used L‐ascorbic acid as the reducing agent to reduce GO. Wang et al. and Stankovich et al.^[^ ^15^, ^16^ ^]^ used dimethylhydrazine and hydroquinone, respectively as the reducing agent to reduce GO. Although, some safe chemicals have been used for the reduction of GO to prepare rGO, unfortunately, most often, the use of toxic reducing agents is one omnipresent drawback associated with the classical chemical reduction method. As an alternative, plant sources‐mediated preparation of rGO, by removing the oxygen functionalities of GO by the phytochemicals present in plant sources, has become attractive because such plant sources‐based reduction methods are environmentally friendly, cheaper, and simpler to execute.^[^ ^17^ ^]^ Wang et al.^[^ ^18^ ^]^ used tea solution for the reduction of GO and got rGO as nanofillers for the fabrication of a bio‐composite with chitosan. Manchala et al. used *Eucalyptus bark* extract for the reduction of GO and get rGO, which was used as anode material for high‐performance supercapacitors.^[^ ^19^ ^]^ Gan et al.^[^ ^20^ ^]^ used sugarcane bagasse extract for the reduction of GO and rGO, which was used as an adsorbent to remove organic dyes. Wijaya et al.^[^ ^21^ ^]^ used kaffir lime peel extract for the reduction of GO and get rGO, which was employed as an adsorbent to remove methylene blue (MB). Rai et al. used ginger extract for the reduction of GO and get rGO, which was employed as anode material for supercapacitors.^[^ ^22^ ^]^ Mahmoud et al used *Ziziphus spinachristi* extract for the reduction of GO and get rGO, which was used as an adsorbent for the removal of MB, an antibacterial agent against gram‐negative bacteria, and as an excellent antioxidant.^[^ ^23^ ^]^ Parthipan et al. utilized *Murraya koenigii* leaf extract for the reduction of GO and get rGO, which was employed as a photocatalyst to remove hazardous organic dyes.^[^ ^24^ ^]^ Mahendran et al.^[^ ^25^ ^]^ used *Bougainvillea glabra* flower extract for the reduction of GO and get rGO, which was utilized as an electrode material to detect Pb^2+^ ions. Bhattacharya et al. used *Aloe vera* extract for the reduction of GO and get rGO, which was employed as an electrode material and as an adsorbent material to remove MB.^[^ ^26^ ^]^ Han et al. used *Cinnamomum verum* (C. verum) bark extract for the reduction of GO and got rGO, which was applied as an anti‐tuberculosis agent.^[^ ^27^ ^]^ Maddinidhi et al.^[^ ^28^ ^]^ used *Terminalia chebula* (T. chebula) seed extract as the reducing agent and Hou et al. used *Lycium barbarum* extract as the reducing agent to reduce GO.^[^ ^29^ ^]^ Mahiuddin et al. used lemon juice for the reduction of GO and get rGO, which was employed as an adsorbent to remove MB.^[^ ^30^ ^]^ Suresh et al. used Cinnamon (*Cinnamomum zeylanicum*) extract for the reduction of GO and get rGO, which was used as an adsorbent to remove organic dyes and as an antioxidant.^[^ ^31^ ^]^

Although much effort has been done, standardizing rGO synthesis is still a difficult endeavor, especially given the influence of the synthesis parameters. Moreover, there is no end to research and we believe that the use of different plant sources to synthesize rGO will enrich the knowledge regarding the plant sources‐based synthesis of rGO and analogous. Therefore, our research focuses on producing rGO using a plant source, which has not been reported yet, and ensures the appropriate use of the obtained rGO as a footstep on the development of plant sources assisted synthetic approach of rGO.

The utilization of green coffee beans for the synthesis of rGO was spurred by the shown capacity of plant sources' functional phytochemicals to reduce GO and to increase functional phytochemicals in green coffee seeds, including phenolic compounds, especially chlorogenic acids; alkaloids, especially caffeine; carbohydrates, especially glucose; lipids, and some volatile and heterocyclic compounds^[^ ^32^, ^33^ ^]^ Moreover, these phytochemicals can actively reduce different metal ions and form their corresponding metal nanoparticles.^[^ ^32^, ^33^, ^34^, ^35^ ^]^ We presume that the functional phytochemicals of coffee beans will also actively reduce GO to produce rGO. To the best of our knowledge, GO has never been reduced to prepare rGO using green coffee beans.

Bangladesh's textile industry is ranked as one of the most polluting industrial sectors.^[^ ^36^ ^]^ The release of colored substances is a cause for alarm, especially in cases when the dye concentration is extremely low (<1 ppm).^[^ ^37^ ^]^ Methylene blue (MB), a cationic dye that is highly soluble in alcohol or water, is the most widely used dye in the textile sector. Inadvertent ingestion of MB might result in methemoglobinemia and other poisoning issues.^[^ ^38^, ^39^ ^]^ Therefore, eliminating MB from an aqueous medium is both extremely difficult and desperately needed. Among various chemical, physical, and biological techniques, adsorption is an important one that has several benefits for large‐scale treatment, including affordability, ease of use, and environmental preservation.^[^ ^38^, ^40^, ^41^, ^42^ ^]^ Although desorption is one of the major obstacles, the adsorption of MB onto graphene derivatives has been extensively documented in the literature.^[^ ^21^, ^43^, ^44^, ^45^, ^46^ ^]^

Therefore, in this study, we illustrate a simple and ecofriendly synthetic protocol of rGO by optimizing the synthetic conditions using green coffee bean as a prominent plant source, where its aqueous extract will be employed as an efficient and sustainable reducing source for the reduction of GO and utilization of the synthesized rGO as a prospective adsorbent to remove MB as one of the model dye.

## 2 Results and Discussion

**Figure 1:**
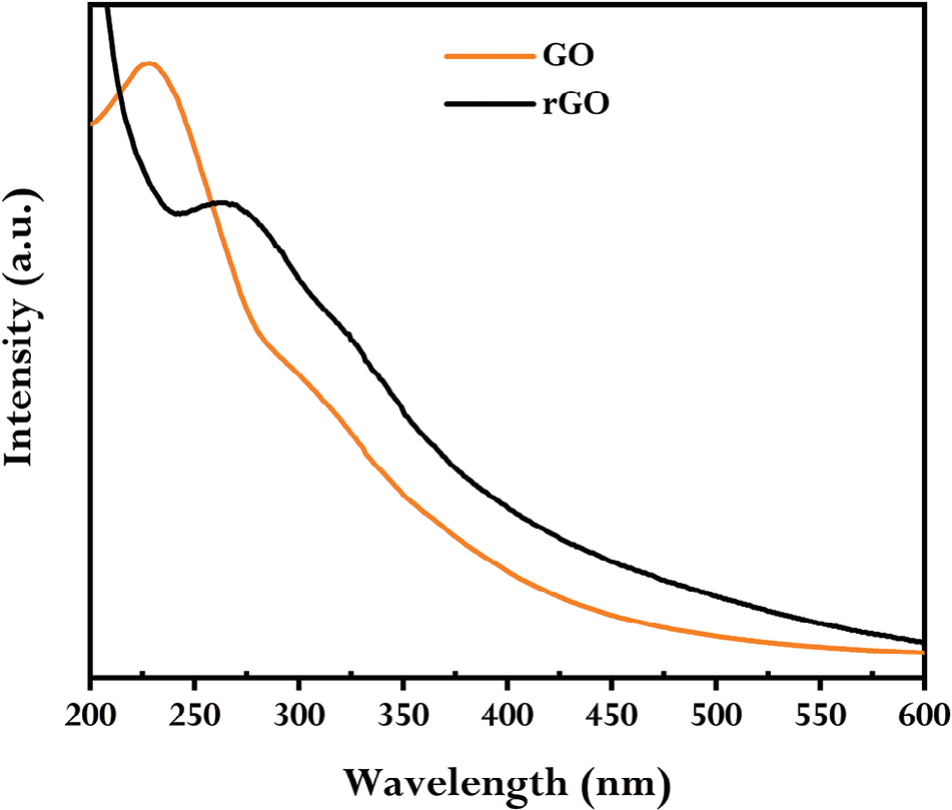
UV–vis spectra of GO and synthesized rGO using GCBE (conditions: GO = 20 mg, GCBE = 20 mL, T = 80 °C, pH = 12, and time = 12 h).

As a feasible and advantageous green‐reducing agent, GCBE was used for the reduction of GO to prepare rGO. The first sign of the reduction of GO and the synthesis of rGO was the reaction mixture's color changing from brownish to deep black. Additionally, using UV–vis spectroscopy, the observation of the distinctive absorption peaks of GO and rGO was the first conformation of the meaningful transformation of GO to rGO (**Figure** **1**).

As seen in Figure 1, rGO has an absorption peak at ∼275 nm in contrast to GO, which has an absorption peak at 231 nm concerning its π–π* transitions. This implies that the majority of GO's oxygen functionalities have been eliminated, and the electronic conjugation—more precisely, C═C—within reduced GO sheets has been restored.^[^ ^12^, ^19^, ^47^ ^]^ UV‐vis spectrophotometer was also employed to track the reaction's development as a function of reaction time, pH, and the amount of GCBE. The time‐dependence UV–vis absorption spectra (Figure S1, Supporting Information) show that the absorption intensity increased and peaks became sharper over time, indicating the gradual generation of rGO, which was consistent with the literature.^[^ ^18^ ^]^ Presumably, a relatively longer reaction time would be suitable for obtaining a quality product. Thus, we consider 12 h as the optimal reaction time for further study. The pH‐dependent UV–vis spectra (Figure S2, Supporting Information) show that the characteristic peak of rGO was unobservable at acidic conditions, but was observable in the characteristic region of rGO at pH 7 and indicated the beginning of the reduction of GO. As the pH increased, the absorption intensity increased and the peaks became sharper, suggesting the rapid reduction of GO at basic conditions. A higher pH is ideal to hasten the phytochemicals' capacity to reduce and produce a high‐quality rGO. In addition, the production of minimum defects in the resultant rGO is encouraged by a reduction procedure carried out in an alkaline environment.^[^ ^17^ ^]^ Thus, we consider the pH value of 12 as optimal for further study. According to the GCBE amount‐dependent UV–vis spectra (Figure S3, Supporting Information), the intensity of the absorption increased and the peaks became sharper as GCBE increased. This indicates that GO was rapidly reduced as a result of the presence of more potent phytochemicals when using a greater amount of GCBE. Thus, 20 mL of GCBE was employed for the further research.

**Figure 2:**
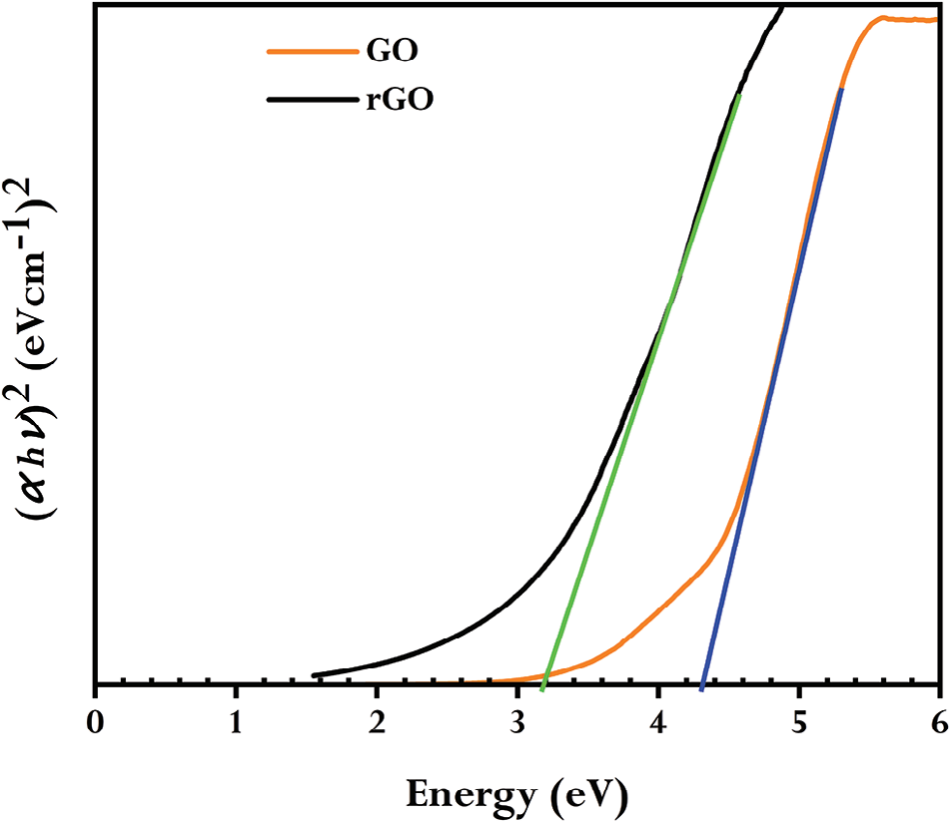
Tauc plot for determining the optical band gap of GO and synthesized rGO using GCBE.

By using Tauc's expression, the energy associated with the optical band gap was computed from UV–vis spectra:

(1) $$\alpha hv\;=B{\left(hv-E_{g}\right)}^{m}$$

where α is the absorption coefficient, *h* is Planck's constant, ν is photon frequency, *B* is a constant, *E_g_* is the optical band gap and *m* = 1/2 for direct allowed transitions. The Tauc optical band gap was determined by extrapolating the linear trend to the x‐axis of the (αhν)^2^ versus the *hv* plot (**Figure** **2**). When GO is reduced by GCBE, the lowering in the optical band gap was observed from 4.25 to 3.2 eV, supporting the formation of rGO. The reduction of GO utilizing the gas‐based hydrazine approach,^[^ ^48^ ^]^ the reduction of GO utilizing mild reagents (glucose, fructose, and ascorbic acid),^[^ ^49^ ^]^ and the reduction of GO using lemon juice in our earlier study^[^ ^30^ ^]^ all showed a similar pattern.

**Figure 3:**
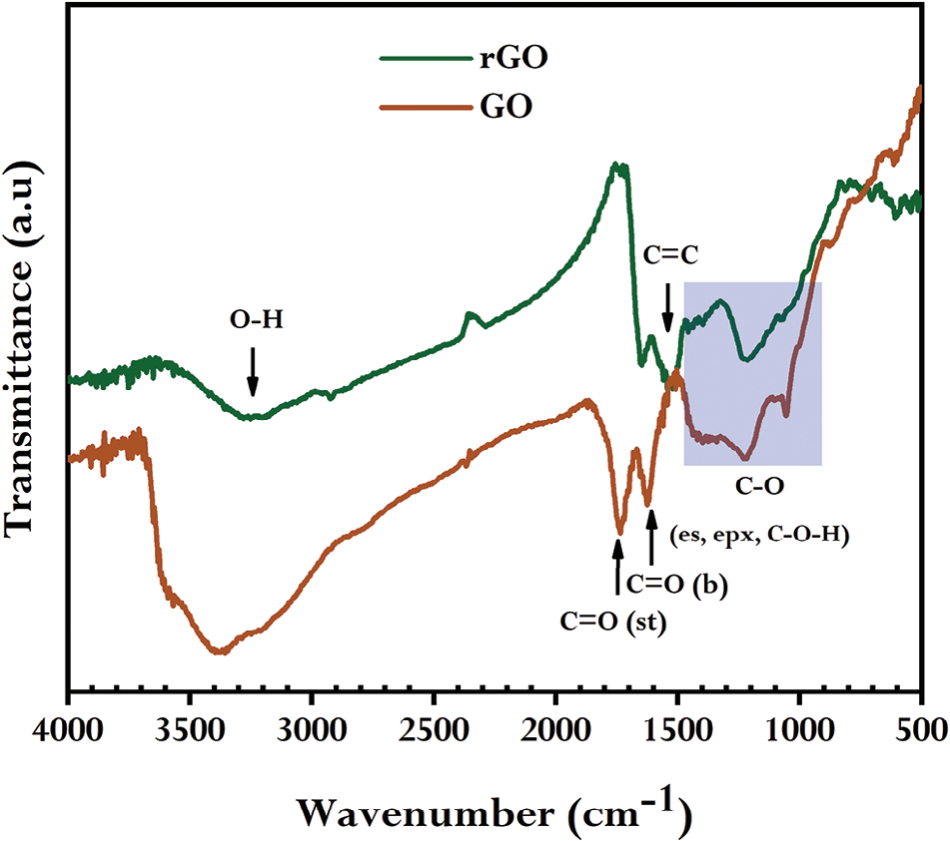
FTIR spectra of GO and synthesized rGO using GCBE (conditions: GO = 20 mg, GCBE = 20 mL, T = 80°C, pH = 12, and time = 12 h).

The functional behavior of GO and synthesized rGO were attained from FTIR spectroscopic analysis. Similar to earlier reports,^[^ ^30^, ^47^ ^]^ a sharp and intense peak of C═O stretching at 1705 cm^−1^ and C═O bending at 1610 cm^−1^; as well as a broad band of O─H stretching at 3447 cm^−1^; and other peaks including C─O─H stretching at 1056 cm^−1^, C─O stretching at 1398 cm^−1^ (epoxy), and 1215 cm^−1^ (alkoxy) were observed from the spectrum of GO. The peak at 1705 cm^−1^ became invisible when GO was reduced using GCBE, however, 1056 and 3447 cm^−1^ peak intensities were significantly decreased (**Figure** **3**), suggesting the elimination of most of the oxygen‐containing functional groups of GO. In contrast, at 1540 cm^−1^, a new and relatively intense peak was observed in the spectrum of rGO attributable to the in‐plane vibration of the C═C skeleton.^[^ ^50^ ^]^ These changes in FTIR spectra understandably recommended the partial and significant reduction of GO would lead to the successful formation of rGO.^[^ ^21^, ^26^ ^]^

**Figure 4:**
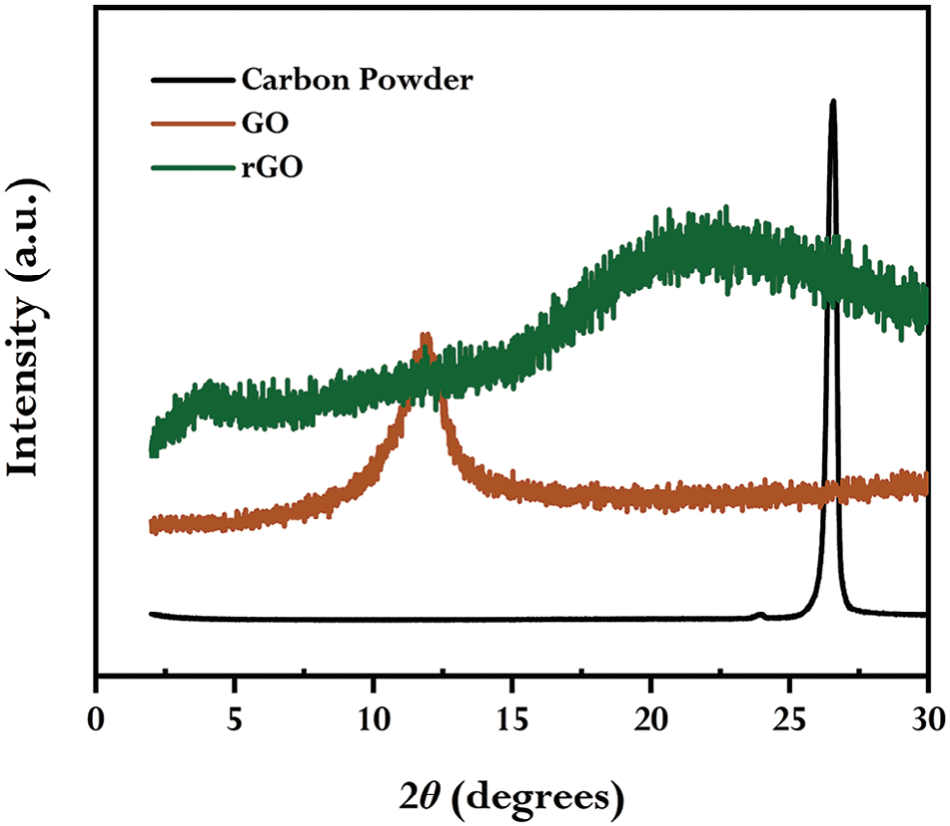
XRD pattern of graphite powder, GO, and synthesized rGO using GCBE (conditions: GO = 20 mg, GCBE = 20 mL, T = 80 °C, pH = 12, and time = 12 h).

Powder XRD analysis was introduced to interpret the crystal structure and interlayer spacing. **Figure** **4** displays the XRD patterns of GO and rGO synthesized using GCBE together with graphite powder.

As described in the literature,^[^ ^3^, ^12^, ^15^ ^]^ the XRD patterns show that graphite powder appears to have a distinctive diffraction peak at 26.6° with an interlayer d‐spacing value of 0.335 nm, which corresponds to the basal reflection (002), whereas GO exhibited the diffraction peak at 12.04° having the interlayer d‐spacing value of 0.734 nm. As GO was reduced by GCBE, the diffraction peak at 12.04° totally vanished, while a wide diffraction peak with an interlayer d‐spacing value of 0.403 nm emerged at 22°, which is the distinctive diffraction area of rGO^[^ ^12^, ^15^, ^51^ ^]^ signifying the successful formation of rGO. The higher interlayer spacing value of GO compared to graphite powder could be attributed to the emergence of oxygen‐containing functional groups and the intercalated water molecules trapped between the graphitic layers. However, upon reduction, the interlayer spacing of rGO was dramatically reduced, demonstrating the effective elimination of GO's oxygen functionalities.^[^ ^18^, ^19^, ^52^ ^]^ According to the earlier report, the broad and weak (002) diffraction of rGO are likely to be reflected in the smaller size (<1 µm) and a short‐order domain and/or disordered stacked sheets of rGO.^[^ ^53^ ^]^

**Figure 5:**
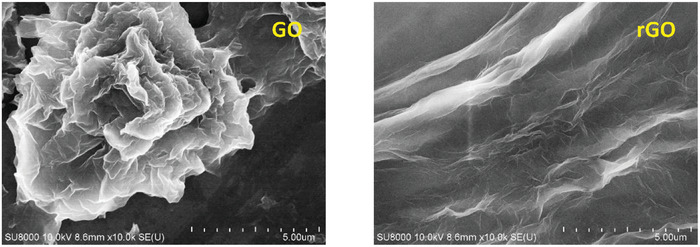
SEM images of GO and synthesized rGO using GCBE (conditions: GO = 20 mg, GCBE = 20 mL, T = 80 °C, pH = 12, and time = 12 h).

The analyses employing electron microscopy were executed to ascertain the surface morphological characteristics of rGO synthesized using GCBE. **Figure** **5** displays the SEM images of GO and synthesized rGO. The SEM image of GO revealed 3D stacked layers of crumpled sheets that were intimately related to one another, whereas, the SEM image of synthesized rGO revealed a few layer of sheets with significant exfoliation and segregation. Similar result was also noticed for previously reported rGO.^[^ ^20^, ^30^, ^51^ ^]^

**Figure 6:**
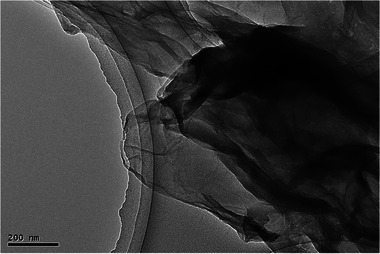
TEM image of synthesized rGO using GCBE (conditions: GO = 20 mg, GCBE = 20 mL, T = 80 °C, pH = 12 and time = 12 h).

The effective formation of exfoliated and segregated layer structures of rGO sheets by the reduction of GO using GCBE is also demonstrated by the TEM image in **Figure** **6**. Typically, the exact number of layers of rGO sheets in the 2D image is not worth considering from TEM analysis, but unobservable graphitic aggregates strongly suggest the substantial formation of exfoliated rGO sheets in a similar way to previously described rGO.^[^ ^19^ ^]^

**Figure 7:**
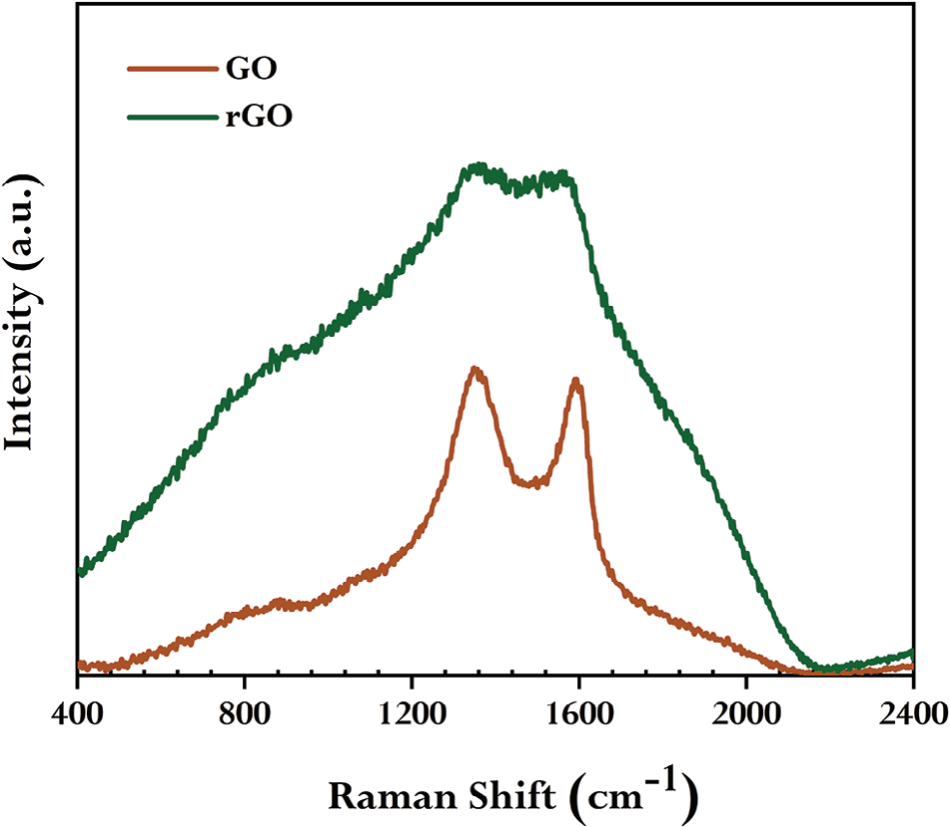
Raman spectra of GO and synthesized rGO using GCBE (conditions: GO = 20 mg, GCBE = 20 mL, T = 80 °C, pH = 12, and time = 12 h).

**Figure** **7** shows the results of a Raman spectroscopy investigation used to assess the qualitative characteristics of carbon structures of synthesized rGO and GO. Similar to the earlier reported works,^[^ ^24^, ^30^, ^51^, ^54^ ^]^ the Raman spectra of GO unveiled two major characteristics of distinctive bands at 1348 and 1589 cm^−1^, which correspond to the D band and the G band, respectively. The D band is associated to the sp3 carbon atoms as a result of the generation of structural defects. After reduction with GCBE, these bands are shifted to 1359 cm^−1^ for the D band and 1566 cm^−1^ for the G band. In addition, the intensity ratio of these bands (*I*_D_/*I*_G_) is another crucial statistic to consider when determining the degree of structural disorder in the carbonaceous materials; larger *I*_D_/*I*_G_ values imply a high degree of structural disorder, whereas lower values imply less.^[^ ^55^ ^]^ According to the *I*_D_/*I*_G_ value, the synthesized rGO has less structural disorder than raw GO, as the *I*_D_/*I*_G_ value of rGO (1.02) is smaller than that of GO (1.04).

**Figure 8:**
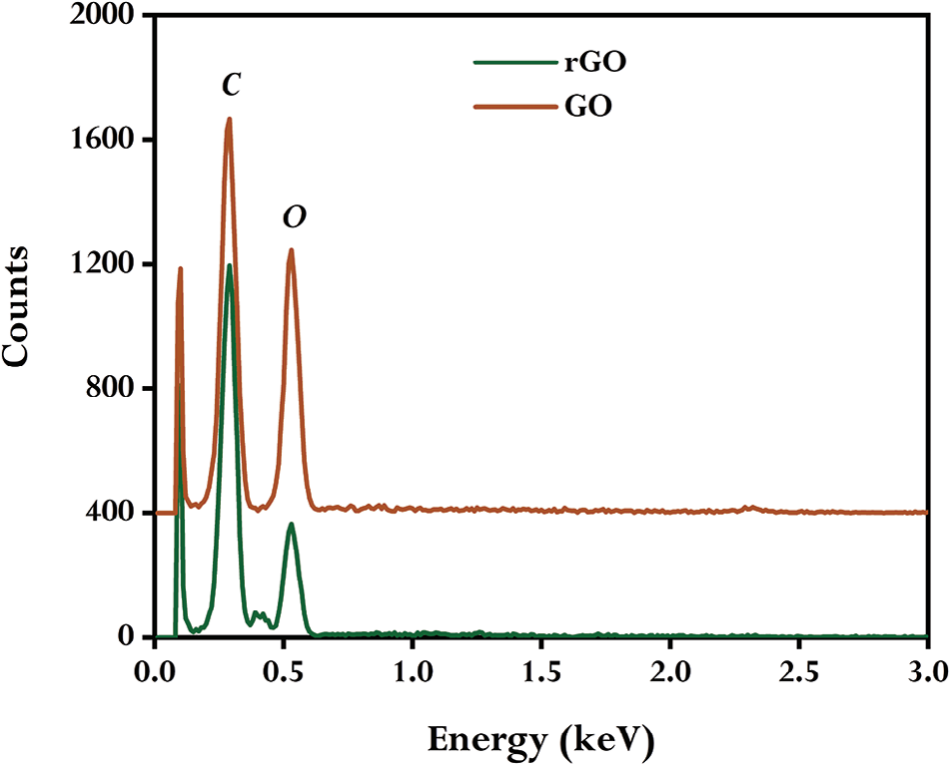
EDX spectra of GO and synthesized rGO using GCBE (conditions: GO = 20 mg, GCBE = 20 mL, T = 80 °C, pH = 12, and time = 12 h).

The EDX spectra of GO and rGO were recorded to identify the formation of the synthesized rGO. EDX spectra of both GO and rGO (**Figure** **8**) revealed carbon and oxygen signals, however the intensity of the signal corresponds to oxygen in rGO is flimsy than that of GO, indicating that GO significantly lost oxygen during its reaction with GCBE, which is also reflected in the higher value of the carbon to oxygen intensity ratio of rGO (3.4) than that of GO (1.39).

**Figure 9:**
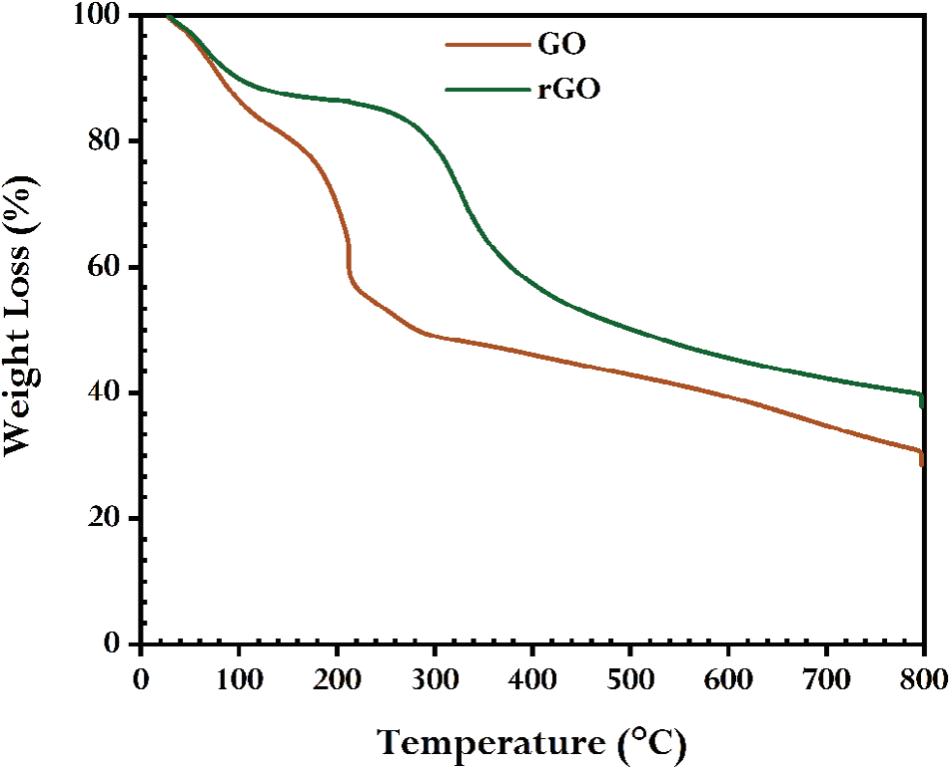
TGA curves of GO and synthesized rGO using GCBE (conditions: GO = 20 mg, GCBE = 20 mL, T = 80 °C, pH = 12, and time = 12 h).

**Figure** **9** illustrates the TGA plot of GO and synthesized rGO that was used to assess the thermal stability of rGO. According to our previous report,^[^ ^30^ ^]^ GO showed weight loss of about 17% up to 120 °C, which was ascertained by removing water molecules that had become trapped between GO sheets, 40% up to 300 °C, which was determined by the burning of functional groups, and 15% after 300 °C, which was brought on by the burning of the GO's carbon skeleton and the further burning of any residual functional groups. Similarly, a significant weight loss of ≈30% was ascertained from the burning of present functional groups in synthesized rGO between 240 and 440 °C, and a total weight loss of ≈60% up to 800 °C indicating significant thermal stability.

### 2.1 Kinetic Modeling

One of the key factors in determining the effectiveness of adsorption is its kinetics. Different scholars have presented various kinetic theories. In the current work, first, we used two crucial kinetic models—pseudo‐first‐order and pseudo‐second‐order kinetics models —to examine the process by which dyes bind to the adsorbent, synthesized rGO. At temperature variations of 298, 308, and 323 K, 15 mg of rGO and 10 mg L^−1^ of MB solution were utilized to conduct the adsorption investigation. The adsorption rates started quickly in each case and then slowed down over the next 20 min. With a removal efficiency of 92 to 96%, the adsorption was at equilibrium after ≈80 min (Figures S4 and S5, Supporting Information). So, for the forthcoming adsorption investigation, we choose 80 min as the equilibrium time.

**Figure 10:**
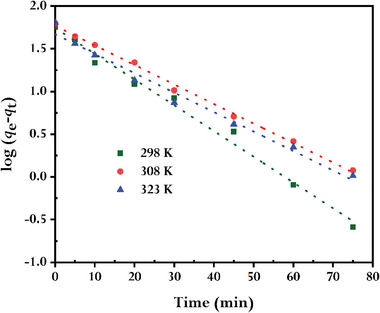
Pseudo–first order kinetics plots for MB adsorbed by synthesized rGO (conditions: CMB0 = 10 mg L^−1^, V = 100 mL; WrGO = 15 mg).

**Figure 11:**
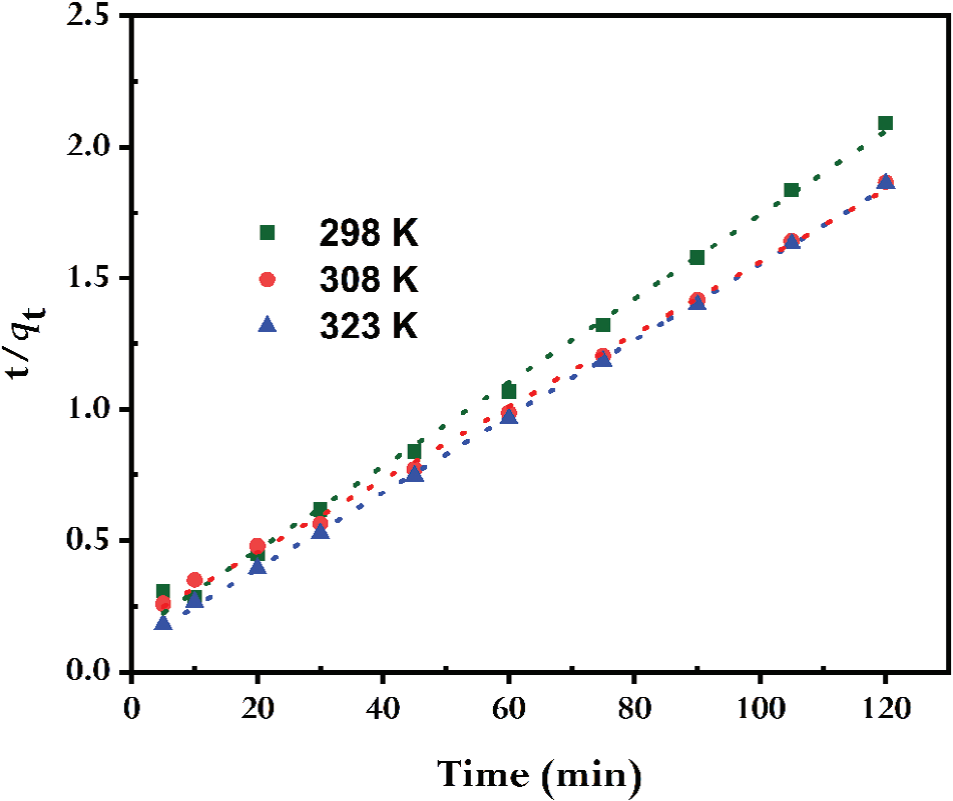
Pseudo–second order kinetics plots for MB adsorbed by synthesized rGO. (conditions: *C*_MB0_ = 10 mg L^−1^, V = 100 mL; *W*_rGO_ = 15 mg).

**Figures** **10** and **11** show the linearized representation of the pseudo‐first‐order and pseudo‐second‐order kinetics models; **Table** **1** lists the pertinent parameters. The correlation coefficients (*R^2^*) obtained from the pseudo‐second‐order kinetic model are higher for each temperature than from the pseudo‐first‐order model, these indicate that the pseudo‐second‐order model fits better to interpret the adsorption process than the other model. Additionally, the estimated *q_e_*’s (62.5, 63.5, 64.3 mg g^−1^) obtained from the pseudo‐second‐order fitting are greater than the *q_e_*’s (55.3, 58.6, 46.7 mg g^−1^) obtained from the pseudo‐first‐order fitting, and are closely comparable with the experimental values (57.0, 63.5, 64.4 mg g^−1^) for each temperature. The nature of MB's adsorption onto prepared rGO is mostly and precisely governed by the chemisorption, according to the kinetics that follow the pseudo‐second‐order model.

**Table 1:** Lists the parameters for MB adsorption on rGO that were determined by several kinetics models.

Models and equations^a)^	Temperature	Parameters			
		*K*_1_ [×10^−2^ min^−1^]	*q*_cal_ [mg g^−1^]	*q*_exp_ [mg g])	*R* ^2^
pseudo‐first order $\log\left(q_{e}-q_{t}\right)=\;\log q_{e}-\frac{k_{1}}{2.303}t$	298 K 303 K 323 K	6.9 5.3 5.2	55.3 58.6 46.7	57.0 63.5 64.3	0.989 0.997 0.983

		*K*_2_ [×10^−3^ g mg^−1.^min^−1^]	*q*_cal_ [mg g^−1^]	*q*_exp_ [mg g^−1^]	*R* ^2^
pseudo‐second order $\frac{t}{q_{t}}=\frac{1}{k_{2}q_{e}^{2}}\;+\frac{t}{q_{e}}$	298 K 303 K 323 K	1.8 1.1 2.1	62.5 72.5 69.0	57.0 63.5 64.3	0.997 0.998 0.999

		*k*_dif_ [mg g^−1.^min^−1/2^]	*R*_i_	*C* [mg g^−1^]	*R* ^2^
Intra‐particle diffusion *q*_*t*_ = *k*_*dif*_ *t* ^1/2^ + *C* $R_{i}=\frac{q_{\mathrm{ref}}-C}{q_{\mathrm{ref}}}\;$	298 K 303 K 323 K	3.8 5.0 3.8	0.54 0.76 0.68	28.7 17.2 21.6	0.757 0.854 0.811
Boyd kinetic *B*_*t*_ = − 0.4977 − ln(1 − *F*) $F\;=\frac{q_{t}}{q_{e}}\;$	298 K 303 K 323 K				0.989 0.997 0.983

^a)^ Conditions: *C_MB0_* = 10 mg L^−1^, *V* = 100 mL, *W_rGO_* = 15 mg.

**Figure 12:**
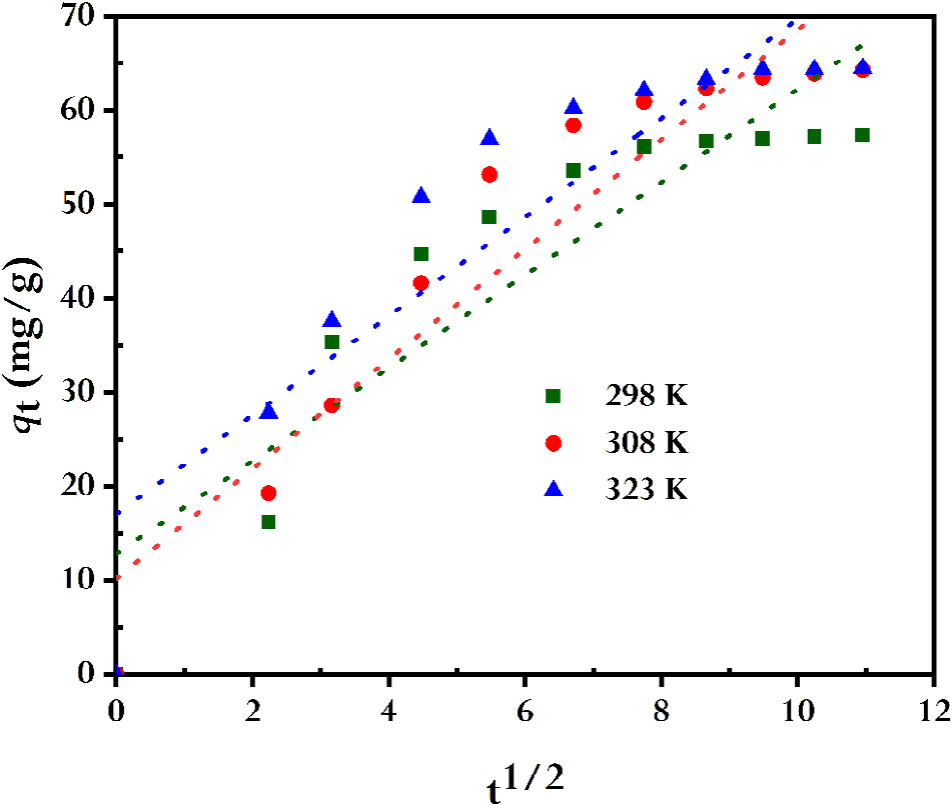
Intra‐particle diffusion (IPD) kinetics plots for MB adsorbed by synthesized rGO (conditions: CMB0 = 10 mg L^−1^, V = 100 mL; WrGO = 15 mg).

**Figure 13:**
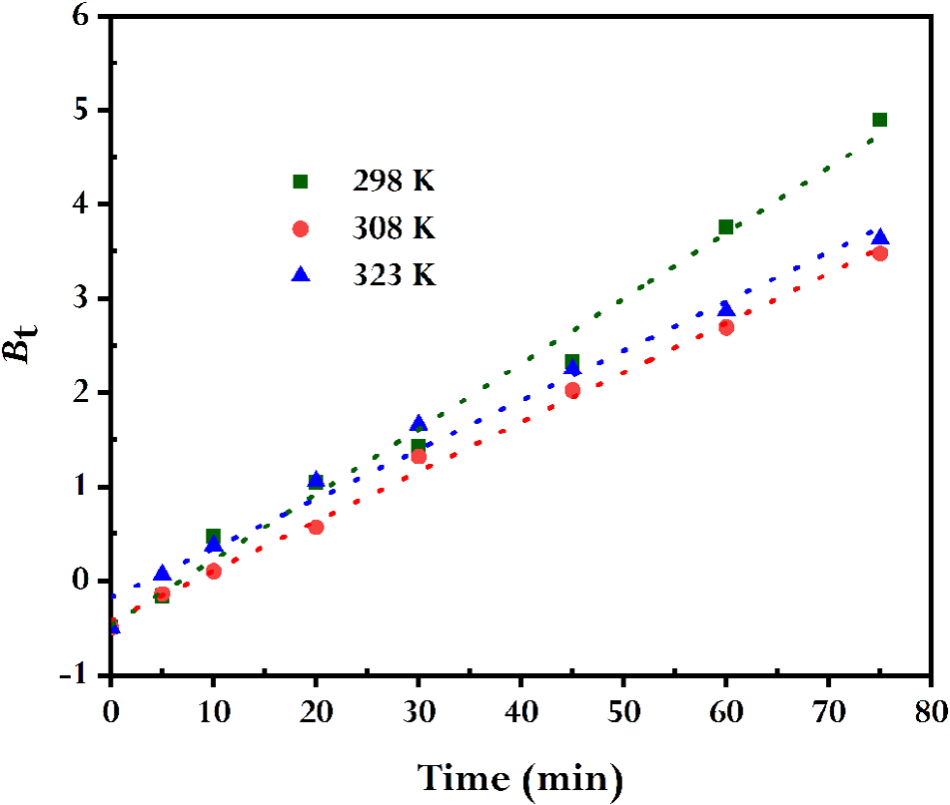
Boyd kinetics plots for MB adsorbed by synthesized rGO (conditions: *C0*MB = 10 mg L^−1^, *V* = 100 mL; *W*rGO = 15 mg).

To pinpoint the mass transport mechanism during the dye adsorption onto rGO, Weber and Morris' intra‐particle diffusion (IPD)^[^ ^56^ ^]^ and the Boyd kinetic^[^ ^57^ ^]^ models are employed. The linearized form of the IPD model is presented in **Figure** **12**. It is clear from the first segment of the curves that dye diffuses through the solution onto the exterior surface of rGO. Meanwhile, the second segment of the curves is associated with gradual adsorption, where intra‐particle diffusion of dye occurs on the surface of the rGO. However, the fact that the line's intercept does not pass through the origin at any temperatures, however, indicates that pore diffusion is not the sole factor determining the rate. According to the findings, the mechanism of dye adsorption onto the green synthetic rGO is complicated, and the actual adsorption process is aided by both intra‐particle diffusion and surface adsorption. Additionally, a visualization of the Boyd kinetic model's linearized version may be seen in **Figure** **13**. It can be shown that the line's intercept does not pass through the origin at any temperature, indicating the movement of MB toward the external surface of synthesized rGO is the rate‐limiting phase in the studied adsorption process. Earlier published works also showed a similar outcome.^[^ ^26^, ^30^ ^]^

### 2.2 Adsorption Isotherm

**Figure 14:**
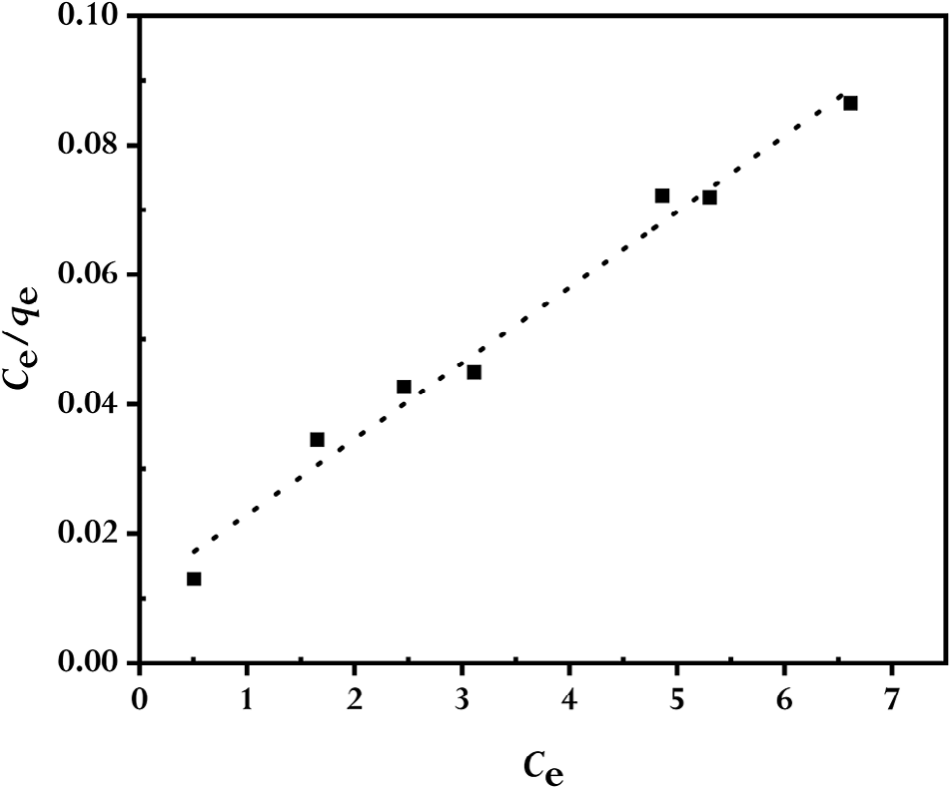
The Langmuir isotherm model for MB adsorbed by synthesized rGO at 298 K (conditions: C0MB = 4 to 16 mg L^−1^, V = 100 mL; WrGO = 10 mg).

**Figure 15:**
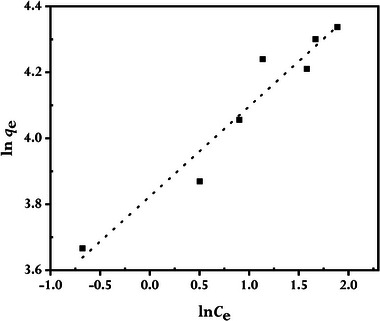
The Freundlich isotherm model for MB adsorbed by synthesized rGO at 298 K (conditions: *C0*MB = 4 to 16 mg L^−1^, *V* = 100 mL; *W*rGO = 10 mg).

**Figure 16:**
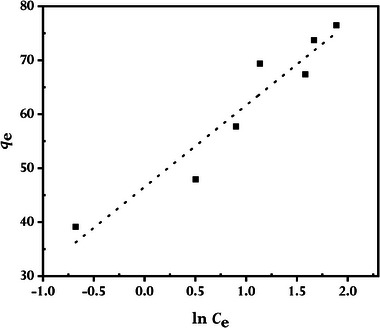
The Temkin isotherm model for MB adsorbed by synthesized rGO at 298 K (conditions: *C0*MB = 4 to 16 mg L^−1^, *V* = 100 mL; *W*rGO = 10 mg).

Adsorption isotherm investigations were conducted to determine the connection between the adsorbate concentrations in the bulk solution (*C_e_*, mg L^−1^) and the quantity of adsorbate absorbed per unit weight of adsorbent (*q_e_*, mg g^−1^).^[^ ^58^ ^]^ Various adsorption isotherm models have been utilized, including the Langmuir and Freundlich adsorption models, which are commonly used to explain how organic dyes adsorb onto graphene derivatives.^[^ ^21^, ^38^, ^39^, ^58^ ^]^ Accordingly, we also employed the Langmuir, Freundlich, and Temkin adsorption isotherm models to assess how MB adsorbed onto rGO synthesized using GCBE. In this investigation, an adsorption duration of 80 min at 298 K temperature was used to test the adsorption of MB at various starting concentrations. The linearized forms of the examined isotherm models are plotted in **Figures** **14**, **15**, **16**; **Table** **2** lists the pertinent parameters with the mathematical expressions.

**Table 2:** Contains the derived constant values and correlation coefficients for several adsorption isotherm models for MB adsorption onto rGO at various temperatures.

Models and equations	Constants	
Langmuir	*q*_m_ [mg g^−1^]	89.3
$\frac{C_{e}}{q_{e}}=\frac{1}{q_{m}b}\;+\frac{C_{e}}{q_{m}};\;\;R_{L}=\frac{1}{1+bC_{0}}$	*R*_L_ *C*_0_ = 4 mg L^−1^ *C*_0_ = 6 mg L^−1^ *C*_0_ = 8 mg L^−1^ *C*_0_ = 10 mg L^−1^ *C*_0_ = 12 mg L^−1^ *C*_0_ = 14 mg L^−1^	0.147 0.103 0.079 0.064 0.054 0.047
	*b* [L mg^−1^]	0.96
	*R* ^2^	0.978
Freundlich	*n*	2.44
$\ln\;q_{e}=\;\ln\;K_{F}+\frac{1}{n}\ln\;C_{e}$	*K*_F_ [mg g^−1^ L mg^−1/n^]	99.7
	*R* ^2^	0.908
Temkin	*b*_T_	57.56
*q*_*e*_ = *B* ln *A*_*T*_ + *B* ln *C*_*e*_; considering $B\;=\frac{\mathrm{RT}}{b_{T}}\;$	*A*_T_ [L g^−1^]	14.58
	*B* [J mol^−1^]	39.43
	*R* ^2^	0.892

Figure 14, 15, 16 of the isotherm plots demonstrate that the Langmuir isotherm models have a much higher correlation coefficient (*R^2^*), indicating that the Langmuir model, as opposed to the Freundlich and Temkin models, better fitted the experimental data, and as a result, the adsorptions of MB are monolayer adsorptions with no inter‐adsorbent interactions. Langmuir adsorption describes, MB monolayer adsorptions mainly on the homogeneous surface of rGO through the interaction of MB and rGO.^[^ ^21^, ^38^, ^39^ ^]^ Additionally, the equilibrium parameter (*R_L_*), also known as the separation factor, was determined; the results are shown in Table 2, where the obtained *R_L_* values are found between 0 and 1, supporting the best fitting of the experimental results by the Langmuir model. Additionally, it was found that the highest amount of MB that could be adsorbed by synthesized rGO per unit weight of total monolayer coverage (*q_m_*) was 89.3 mg g^−1^. This value was in agreement with the experimental finding and demonstrated the validity of the Langmuir isotherm model toward the adsorption of MB onto synthesized rGO.

In comparison to other graphene‐based adsorbents that have been reported, synthesized rGO from this work has a *q_m_* value (89.3 mg g^−1^) that is greater than exfoliated GO (17.3 mg g^−1^),^[^ ^59^ ^]^ non‐functionalized oil plam waste‐derived rGO (50.07 mg g^−1^),^[^ ^46^ ^]^ and rGO‐based hydrogel (7.85 mg g^−1^)^[^ ^58^ ^]^ but slightly lower than the nanocomposite of *β*‐cyclodextrin/magnetic GO (94 mg g^−1^)^[^ ^60^ ^]^ and interconnected rGO (106.0 mg g^−1^),^[^ ^44^ ^]^ indicating that rGO produced via GCBE would be a strong contender for removing MB.

### 2.3 Effect of Mass of rGO on the Adsorption Process

**Figure 17:**
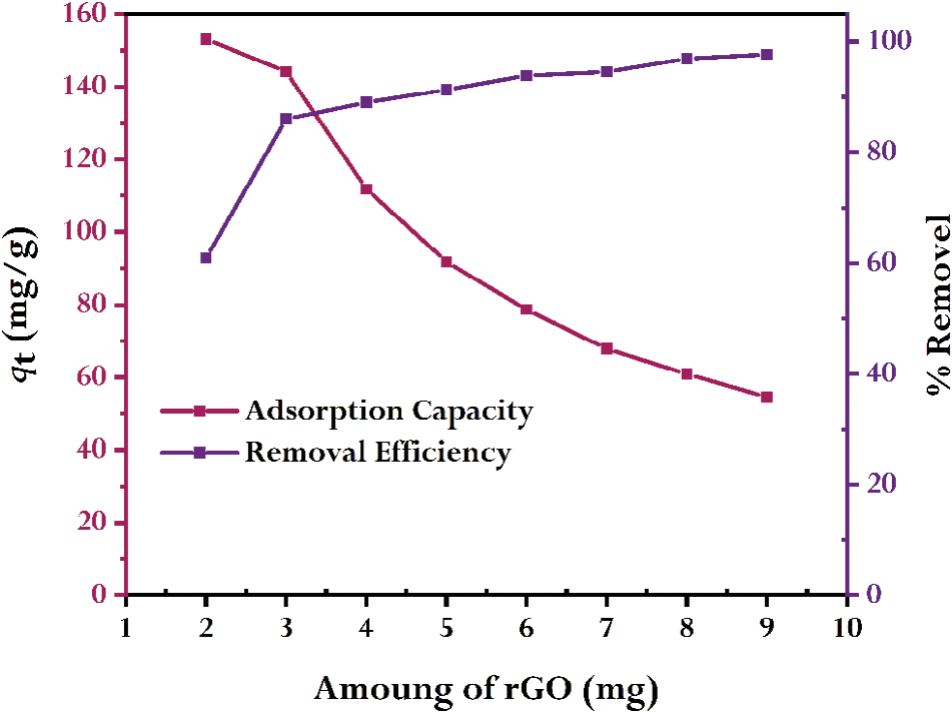
Effect of rGO mass on the adsorption onto rGO (conditions: C0MB = 10 mg L^−1^, V = 50 mL, T = 298 K).

The adsorption of MB onto rGO is influenced by several factors, including the mass or amount of rGO used in the process. **Figure** **17** displays the outcome of this investigation, which was carried out using 2 to 9 mg of rGO at 298K with the constant of the other parameters. The removal efficiency rises from 60.9 to 97.7% when the mass of rGO utilized for adsorption is increased from 2 to 9 mg. As a result, more MB is adsorbed when there is a greater mass of rGO since there is more surface area and active sites for MB to connect to. In contrast, when the mass of rGO increases, the adsorption capacity falls from 153.1 to 54.6 mg g^−1^. This indicates that there are a lot more rGO active sites available even after all of the MB has been adsorbed since the number of rGO active sites is far more than the number of MB molecules, which is thought to be the cause of the reduced adsorption capacity.

### 2.4 Effect of Initial Concentration of MB on the Adsorption Process

**Figure 18:**
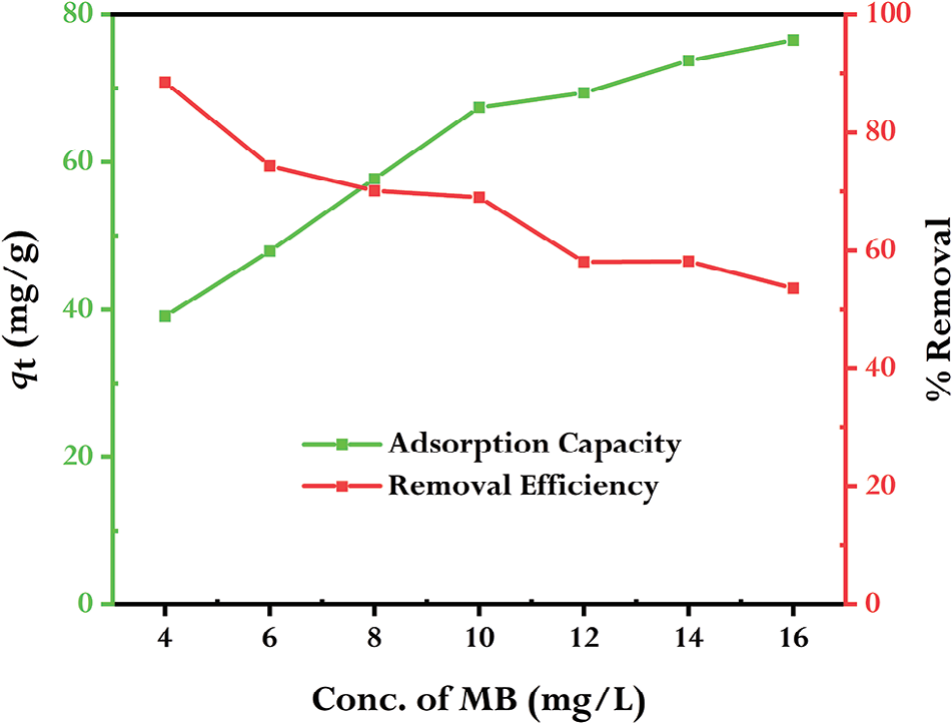
Effect of the initial MB concentration on the adsorption onto rGO (conditions: WrGO = 10 mg, V = 100 mL, T = 298).

The initial concentration of MB in the solution is an important parameter that significantly influenced the adsorption of dye molecules onto the rGO surface. **Figure** **18** depicts the results of investigations done at 298 K using the initial dye concentrations of 4, 6, 8, 10, 12, 14, and 16 mg L^−1^ of 100 mL of MB solution with 10 mg of rGO. As the initial concentration of MB increases, the removal efficiency of rGO decreases from 88.5 to 53.6%. This is because, as the initial concentration becomes very high, the available adsorption sites can become saturated, and the removal efficiency drops. In contrast, the adsorption capacity of rGO increases from 39.2 to 78.5 mg g^−1^ when the starting concentration of MB rises. Due to the fact that there are more dye molecules in the solution, there is a larger chance that they will come into touch with and bind to available adsorption sites on the rGO surface at a higher initial concentration.

It was commonly known how MB adsorbs onto rGO.^[^ ^38^, ^40^ ^]^ As a result, we also presented the presumable mechanism behind the MB adsorption process on rGO that was synthesized with the use of green coffee bean extract. Briefly, the primary building blocks of rGO are C─C and C═C, with traces of C═O. However, MB has sulfur, C═C, and a distinct type of nitrogen atom (having a lone pair electron and a positive charge). Therefore, H‐bonding between neutral nitrogen and sulfur atom and hydrogen atom of the various functional groups of rGO, electrostatic attraction between oxygen of rGO and positive nitrogen of MB, and π─π interaction between C═C of rGO and MB may control the adsorption process of MB onto environmentally favorable produced rGO.

## 3 Conclusion

Using green coffee bean extract (GCBE) as a green and safe reducing agent, the work offers a unique synthetic method for producing reduced graphene oxide (rGO) by optimizing reaction parameters including reaction duration, pH, and extract concentration. The effectiveness of the synthesized rGO in removing methylene blue, a common dye pollution, from aqueous solutions was assessed. Various spectroscopic, XRD, electron microscopic, and TG analysis investigations supported the formation of rGO through the regeneration of conjugated systems by the efficient elimination of the functional groups of GO and obtained exfoliated rGO having a few layers of sheets. The synthesized rGO has a maximal adsorption capacity of 89.3 mg g^−1^ and demonstrates remarkably quick dye adsorption. The pseudo‐second‐order kinetic model and the Langmuir isotherm model provide the best explanation of the adsorption process, the nature of adsorption, and the affinity between rGO and MB. This model suggests that the chemisorption of MB onto the homogeneous surface of rGO through hydrogen bonding, electrostatic attraction, and π─π interactions may be the most likely mechanism for the adsorption process. Overall, the study indicates that the suggested method provides a sustainable and ecologically acceptable way of producing rGO and removing organic dyes. By utilizing a natural plant extract, the study contributes to the growing field of green processes for rGO preparation, which aims to try to minimize the utilization of harsh chemicals and energy‐intensive processes. The research ultimately underscores the importance of sustainable and eco‐friendly approaches in material synthesis and environmental remediation.

## 4 Experimental Section

### Sampling and Extraction of Green Coffee Bean

Green coffee beans (GCB) were purchased from the super shop in Yonezawa, Yamagata prefecture, Japan, and thoroughly washed with deionized distilled water (DDW) and dried completely using a vacuum dryer. The dried green coffee beans were ground to a fine powder. The extract was obtained by adding 100 g of fine powder to 400 mL of DDW and heating at 80 °C for 3 h. The extract (GCBE) was collected through filtration (using Whatman's filter paper) followed by centrifugation (10000 rpm, 10 min) and finally stored at 4 °C for further use.

### Materials

Graphite powder purchased from Sigma‐Aldrich (St. Louis, MO, USA). Sodium borohydride (NaBH_4_), Sodium nitrate (NaNO_3_), Hydrogen peroxide (H_2_O_2_), Sulfuric acid (H_2_SO_4_), Methylene blue (MB), and Sodium Hydroxide (NaOH) were purchased from Kanto Chemical Co. Inc. (Tokyo, Japan). Potassium permanganate (KMnO_4_) was obtained from Tokyo Chemical Industry Co. Ltd. (Tokyo, Japan). All of the reagents were of analytical grade and were used directly without further purification.

### Synthesis of Graphene Oxide (GO)

GO was prepared using a modified Hummer's method as described in our previous report.^[^ ^30^ ^]^ Briefly, 2 g of graphite powder was mixed with 80 mL of concentrated sulfuric acid under ice bath conditions by maintaining the temperature below 0–5 °C, after 30 min 2 g of sodium nitrate was added, and continued stirring for 1 h. Then 12 g of potassium permanganate (KMnO4) was added slowly to the mixture and controlled temperature below 10 °C with the next 1 h stirring. Subsequently, the mixture was transferred to an oil bath, and the temperature was maintained at 40 °C with constant stirring for 2 h, followed by the addition of 80 mL of water raising the temperature to 90 °C and continued stirring for another 1 h. After that mixture was stirred at room temperature overnight. To control the pH of the reaction medium and to terminate the reaction 180 mL of water followed by 20 mL of H_2_O_2_ was added to the above reaction mixture. The color of the suspension changed from brown to light yellow indicating the oxidation of graphite to GO. After centrifugation of solution at 10 000 rpm, GO was washed with 2 m HCl (to remove excess of metal ions) and distilled water several times. The resultant GO was vacuum dried at 60 °C for 24 h and then stored for use in synthesizing reduced graphene oxide (rGO).

### Synthesis of rGO Using Extract of Green Coffee Bean

20 mg GO was mixed with an appropriate volume of GCBE (10–20 mL) by sonication, then added water to obtain a total volume of 50 mL, and adjusted the pH (6–12) of the mixture using 1 m aqueous NaOH. Then the mixture was stirred in an oil bath at 80 °C at different times (up to 12 h). The resulting black product was then separated using centrifugation and washed four times using water. The product was then vacuum dried for 24 h at 60 °C before being stored at 25 °C.

### Measurements

UV–vis spectroscopic analysis was carried out on a DR 5000 (HACH, Colorado, USA) spectrometer within the range of 200–800 nm with a resolution of 1 nm. FTIR spectra were recorded using a KBr pellet on a JASCO (Tokyo, Japan) FT/IR‐460 plus spectrometer at a scan rate of 4 cm^−1^ s^−1^ ≈25 C. SEM measurements and EDX analysis were conducted on a Hitachi (Tokyo, Japan) SU‐8000 microscope at accelerating voltages of 10 and 15 kV. TEM measurement was conducted on a JEOL TEM‐2100F field emission electron microscope. XRD analysis was conducted on a Rigaku (Tokyo, Japan) MiniFlex 600 diffractometer with Cu‐Kα radiation. Raman spectra were recorded on a JASCO (Tokyo, Japan) RPM‐320 spectrometer with a laser of 532 nm using powder samples. TGA was carried out on a Seiko Instruments (Tokyo, Japan) TG/DTA 6200 (EXSTER6000) at a heating rate of 10 °C min^−1^ under N_2_.

### Preparation of Methylene Blue (MB) Solutions

A 1000 mg L^−1^ stock solution of MB was prepared by dissolving MB powder in de‐ionized (DI) water. The stock solution from MB was diluted to 10 mg L^−1^ to prepare the solution that was utilized in the experiment.

### The Experiment of MB Adsorption on rGO

An adsorption study was conducted based on our previous work.^[^ ^30^ ^]^ In brief, by introducing 15 mg rGO to 100 mL of MB solution at a concentration of 10 mg L^−1^ in a 200 mL round bottom flask, an evaluation of the adsorption kinetics and the influence of contact time of MB adsorption onto rGO was conducted. A magnetic stirrer was used to agitate the resultant mixture at 150 rpm for up to 2 h at three different temperatures (25, 35, and 50 °C). The rGO and MB were separated at the specified time by centrifugation at 10,000 rpm for 5 min. The final concentrations of MB were analyzed by UV–vis spectrophotometer at 665 nm. Similarly, the adsorption isotherms were evaluated from a batch experiment at 25 °C in which 10 mg of rGO was added to 100 mL of MB solutions at various concentrations ranging from 4 to 16 mgL^−1^ (step size: 2 mgL^−1^). In addition, 50 mL of MB solution with a concentration of 10 mg L^−1^ was used to measure the removal of MB as a function of rGO mass by mixing additional masses of rGO (2–9 mg, step size: 1 mg).

The following equation was employed to calculate the adsorption capacity of rGO:

(2) $$q_{t}=\;\frac{\left(C_{0}-C_{t}\right)V}{W}$$

where *C*_0_ and *C_t_* are the concentrations (mg L^−1^) of MB at initial and at time t, respectively; *V* represents the volume of the solution (L); W is the adsorbent mass (g).

The following equation is employed to calculate the removal efficiency (RE%):

(3) $$RE\%\;=\;\frac{\left(C_{0}-C_{e}\right)}{C_{0}}\;X100$$

where *C_e_* represents the equilibrium concentration.

## Conflict of Interest

The authors declare no conflict of interest.

## Supporting information

Supporting Information

## Data Availability

The data that support the findings of this study are available from the corresponding author upon reasonable request.
