# Supplementary material for: Green Coffee Bean Extract Assisted Facile Synthesis of Reduced Graphene Oxide and Its Dye Removal Activity
Source: Glob Chall. 2023 Dec 21;8(1):2300247. doi: 10.1002/gch2.202300247 (PMC10784199; doi:10.1002/gch2.202300247)
Supplement: Supplementary file 1 — Supporting Information [file GCH2-8-2300247-s001.pdf]

Global Challenges

Open Access

Supporting Information

for *Global Challenges.*, DOI 10.1002/gch2.202300247

Green Coffee Bean Extract Assisted Facile Synthesis of Reduced Graphene Oxide and Its Dye Removal Activity

*A.B.M. Nazmul Islam, Prianka Saha, Md. Emran Hossain, Md. Ahsan Habib, Kaykobad Md. Rezaul Karim and Md. Mahiuddin**

<u>Supplementary Information</u>

Green Coffee Bean Extract Assisted Facile Synthesis of Reduced Graphene Oxide and its Dye Removal Activity

A.B.M. Nazmul Islam, Prianka Saha, Md. Emran Hossain, Md. Ahsan Habib, Kaykobad Md. Rezaul Karim, and Md. Mahiuddin*

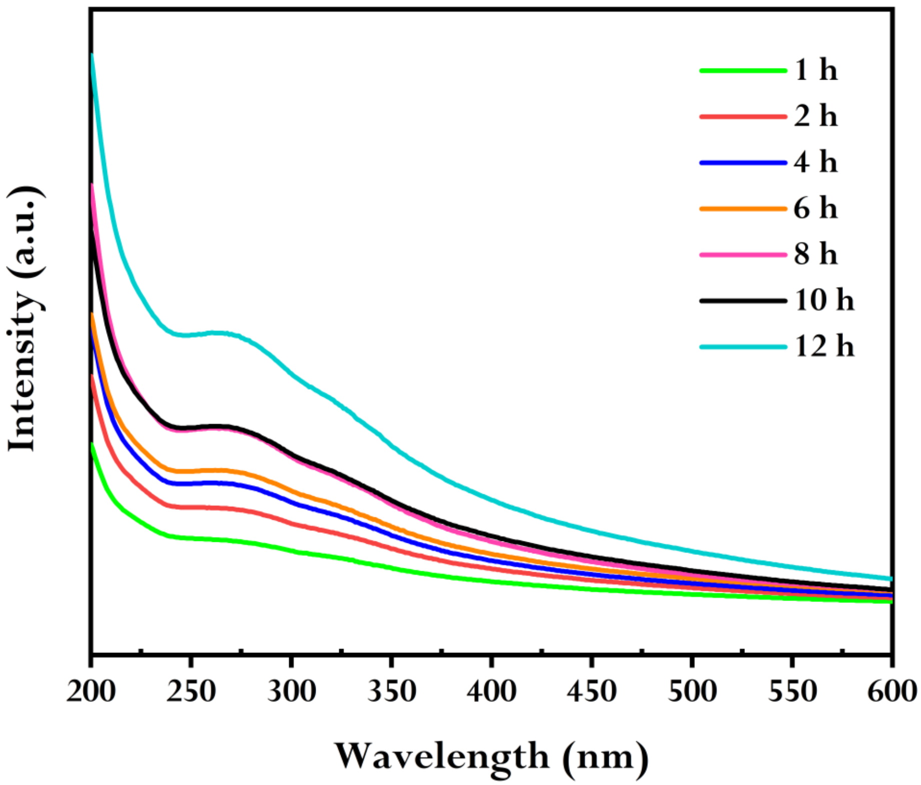

Figure S1. Time-dependent UV-vis spectra of the reaction mixture of the reduction of GO to rGO using GCBE (conditions: pH = 12.0, temperature = 80 °C, GCBE = 15 mL).

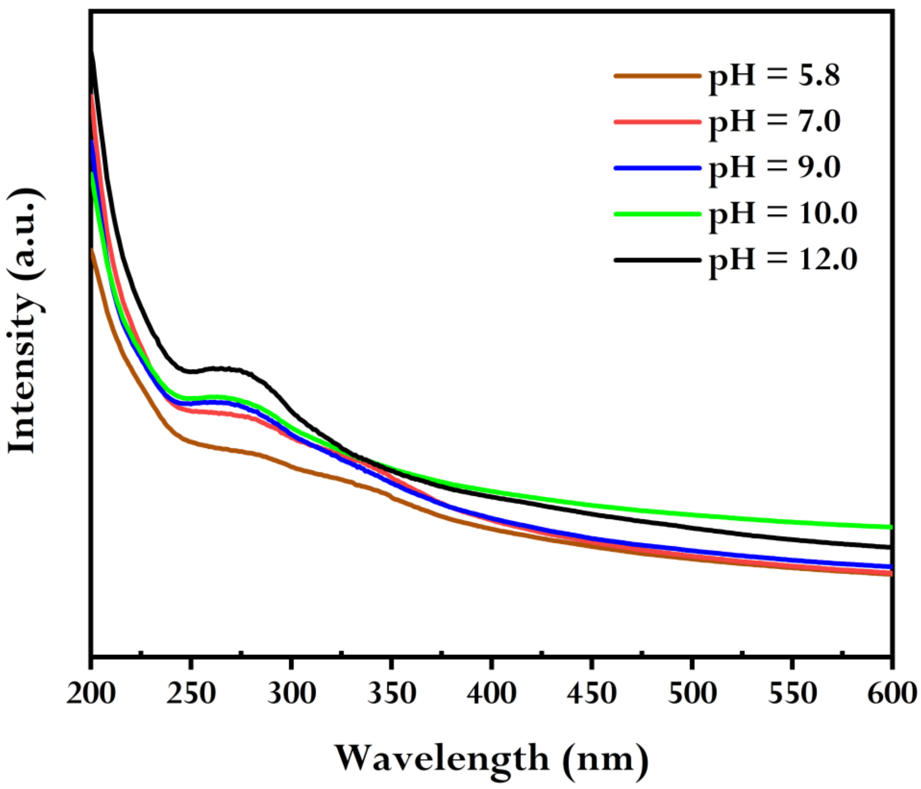

Figure S2. pH-dependent UV-vis spectra of the reaction mixture of the reduction of GO to rGO using GCBE (conditions: time = 12 h, temperature = 80 °C, GCBE = 15 mL).

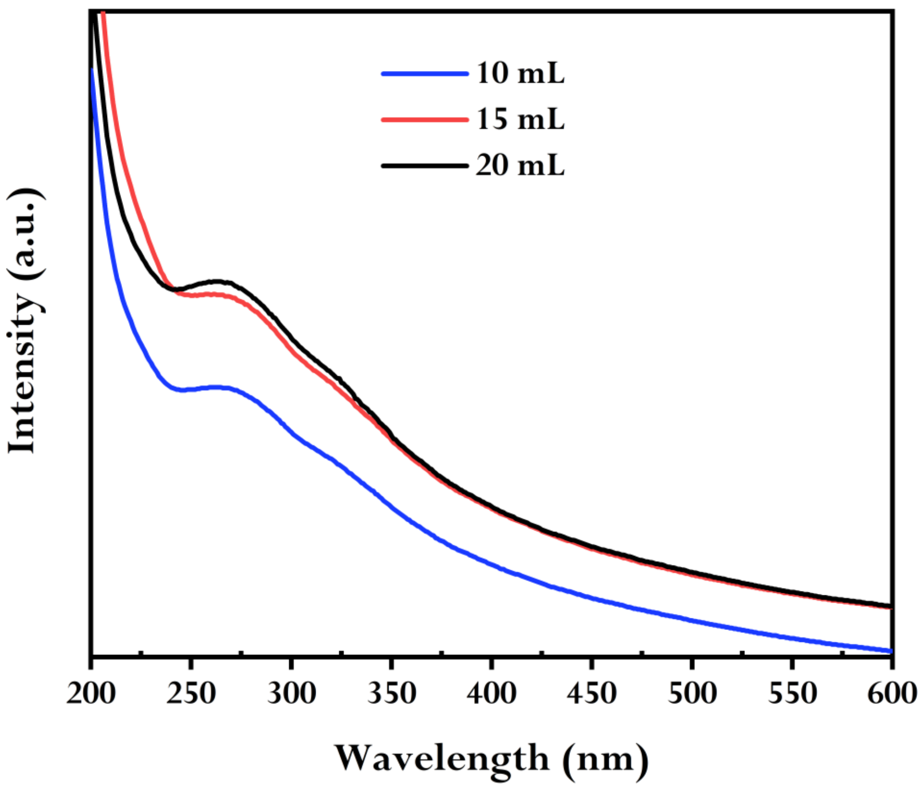

Figure S3. UV-vis spectra of the reaction mixture of the reduction of GO to rGO using different amounts of GCBE (conditions: pH = 12, time = 12 h, and temperature = 80 °C).

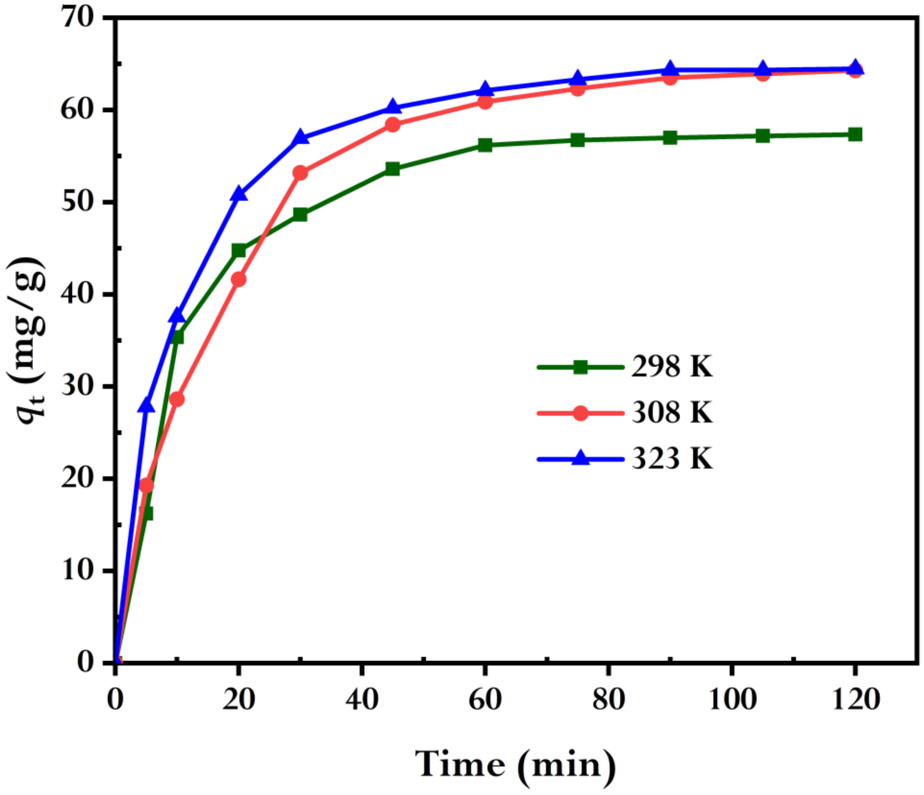

Figure S4. Time-dependence adsorption capacity of MB onto green synthesized rGO (conditions: C_{MB0} = 10 mg/L, V = 100 mL; W_{rGO} = 15 mg).

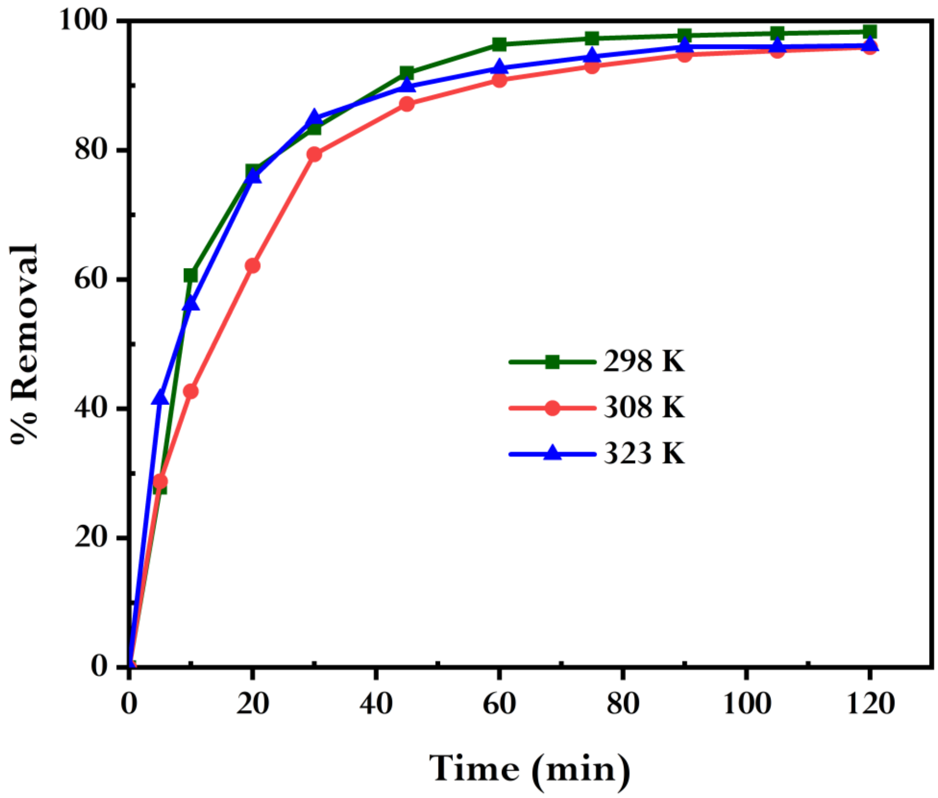

Figure S5. Time-dependence removal efficiency of MB onto green synthesized rGO (conditions: C_{MB0} = 10 mg/L, V = 100 mL; W_{rGO} = 15 mg).
